# The journey of thesis supervisors from novice to expert: a grounded theory study

**DOI:** 10.1186/s12909-019-1739-z

**Published:** 2019-08-22

**Authors:** Leila Bazrafkan, Alireza Yousefy, Mitra Amini, Nikoo Yamani

**Affiliations:** 1 Clinical Education Research Center, Shiraz University of Medical Sciences, Shiraz, Iran; 2 Department of Medical Education, Medical Education Research Center, Isfahan University of Medical Sciences, Isfahan, Iran

**Keywords:** Qualitative research, Medical sciences faculty, Grounded theory, Expertise, Thesis supervision

## Abstract

**Background:**

Supervision is a well-defined interpersonal relationship between the thesis supervisors and their students. The purpose of this study was to identify the patterns which can explain the process of expertise attainment by thesis supervisors. We aimed at developing a conceptual framework/model to explain this development based on the experience of both students and supervisors.

**Methods:**

We have conducted a qualitative grounded theory study in 20 universities of medical sciences in Iran since 2017 by using purposive, snowball sampling, and theoretical sampling and enrolled 84 participants. The data were gathered through semi-structured interviews. Based on the encoding approach of Strauss and Corbin (1998), the data underwent open, axial, and selective coding by constant comparative analysis. Then, the core variables were selected, and a model was developed.

**Results:**

We could obtain three themes and seven related subthemes, the central variable, which explains the process of expertise as the phenomenon of concentration and makes an association among the subthemes, was interactive accountability. The key dimensions during expertise process which generated the supervisors’ competence development in research supervision consisted maturation; also, seven subthemes as curious observation, evaluation of the reality, poorly structured rules, lack of time, reflection in action, reflection on action, and interactive accountability emerged which explain the process of expertise attainment by thesis supervisors.

**Conclusions:**

As the core variable in the expertise process, accountability must be considered in expertise development program planning and decision- making. In other words, efforts must be made to improve responsibility and responsiveness.

## Background

Supervision is a well-defined term in the interpersonal relationship between thesis supervisors and students. A supervisor is designated to assist the student’s development in terms of their research project [1–3]. Faculty members supervise the students because qualified supervision leads to success on the part of the student, and it has moral, reputational, and financial outcomes for the institution. Supervisors are expected to train students to gain competence in areas such as specialist skills, generalist skills, self-reliance skills, and group/team skills [4]. Expertise is derived from the three essential elements of knowledge, experience, and the ability to solve problems in society [5–7]. .According to Dreyfus, acquisition of expertise or practical wisdom represents a higher level of “self-actualization.” At this point, one reaches a level in which they can flourish in their talents and abilities. This enables the teachers to function in scientific communities and multicultural environments [7].

Wiscer has identified three stages in the thesis supervision process and describes the duties of the supervisors in each of them [8]. Pearson and Brew state that maturation in specialist skills, generalist skills, self-reliance skills, and group/team skills are the major areas that need to be promoted in the student. Moreover, these are the generic processes in which the supervisors should be involved for efficacious supervision if they aim to help the students develop in various institutional, disciplinary and professional settings; acquire appropriate expertise and features needed for employment; and make an outline of what might form a flexible professional development program for supervisors in this setting [3]. Vereijken et al. emphasized novice supervisors’ approaches to reach expertise in supervision and explained the relationship between practice and dilemmas among novice supervisors [9].

.Despite the importance of expertise in higher education and particularly research supervision, research abilities are not considered as one of the priorities in the employment of the academic staff. Furthermore, the newly employed faculty members are often involved in teaching, administrative tasks, and services in health care; this inhibits them from expertise attainment in other aspects such as research supervision [10–12]. In this regard, Malekafzali believes that in the area of research activities, the faculty members have serious weaknesses in defining the problem, choosing the appropriate method for research, analyzing the data, interpreting the results, and publishing scientific articles. Besides, there is a lack of coherent and compiled training programs which can enhance their research capabilities [13].

One of the most important factors contributing to the thesis and research quality is the process of developing expertise in supervisors’ research supervision. Most studies in our country have focused on research abilities during the research, and fewer studies have focused on the process of expertise acquisition in thesis supervision, and no actual model has been proposed for this [11–13]. The quantitative researches could not explain exactly how and through which process the faculty members, as thesis supervisors, become experts in thesis supervision since the expertise process is multi-factorial and has many unknown aspects. Considering the effective role of qualitative research in clarifying ambiguous and unknown aspects, we chose the grounded theory approach for this study [14–17]. This theory will be used when the investigator intends to determine the patterns of actions and social interactions needed for the development of expertise by specific groups of people in a specific setting [17, 18].

### Objectives

In this study, we aimed to identify the themes that explain the expertise development process among thesis supervisors in Iran, and also to develop a conceptual framework/model to explain this development based on the experience of both students and supervisors.

## Methods

### Setting

This study was carried out in 20 universities of medical sciences with different ranks in Iran because universities are the places where supervisors and students interact purposefully to discourse the needs of experts on specific occasions and in specific conditions. In these universities, different students study with various disciplines. There are three types of universities in Iran. Type 1 universities are the ones with the most facilities, faculties, research presentations, international collaborations, and scientific outcomes. The second rank belongs to type 2, and the one with the least mentioned qualities is type 3 universities. All three types of universities were included in this study. In all these courses, writing a thesis is one of the requirements with the same role and regulation. The majority of the students in this research project were in the late stages of both undergraduate and postgraduate educational programs within the same function and regulation.

### Study design

We conducted this qualitative study based on a grounded theory approach in a systematic form [17, 18]. Grounded theory is a symbolic interaction which is derived from systematic data collection during the research process. In this strategy, collecting and analyzing data and the theory derived from the data have a close association [17, 19]. The investigator’s purpose in using grounded theory is to describe and clarify a phenomenon in the social condition and to identify the essential processes working within [17].

### Participants

In this study, 84 subjects including 56 faculty members of medical sciences, 20 undergraduate and postgraduate students (medical students, MS of Science, Ph.D. and residents), and eight managers in the field of research supervision participated. Using purposive sampling, snowball sampling with maximum variation, we selected the participants from a variety of academic ranks with different work experiences, as the key informants in thesis supervisors. Then, to continue the sampling, we used theoretical sampling and data saturation. The inclusion criterion was 5 years of work experience in thesis supervision, and the exclusion criterion was the unwillingness to participate in the study. Firstly, we collected data in Shiraz University with the help of a research supervisor who is known for his high quality of supervision and then data gathering was initiated in the university of Isfahan. There were 34 key informants from the two universities and 22 individuals from other universities. Students were selected based on their willingness to participate.

Theoretical sampling was used next to develop the tentative theory. The basis for theoretical sampling was the queries that emerged during data analysis. At this stage, the researcher interviewed the supervisor, administrators, and students. Theoretical sampling facilitated in verifying the supervisors’ responses and credibility of categories and resulted in more conceptual density. Data saturation was obtained when no new data emerged in the last five interviews. Therefore, data gathering by interviews was terminated.

### Data collection

We collected the data primarily by semi-structured interviews from September 2017 to September 2018. The participants were recognized with unknown codes based on their field of work and setting, and each participant was interviewed in one or two sessions. Having obtained the participants’ informed consent, we recorded the interviews and they were transcribed verbatim immediately. The interviews began with open-ended general questions such as, “What did you experience during research supervision?” and then the participants were asked to describe their perceptions regarding their expertise process. Leading questions were also used to deeply explore the conditions, processes, and other factors that participants recognized as significant issues. The interview was based mostly on the questions which came up during the interview. On average, each interview lasted for an hour, during which field notes and memos were taken. At the end of each session, the participants were asked to give an opinion on other important topics which did not come up during the interview, followed by data collection and analysis which are simultaneously done in grounded theory; analytic thought and queries that arose from one interview were carried to the next one [20].

The data were also collected by unstructured observations of the educational atmosphere in the laboratory, and the faculty member and students’ counseling offices. These observations lasted 5 weeks, during which the faculties and students’ interactions and the manner of supervision were closely monitored. The observation was arranged to sample the maximum variety of research supervisor activity for some faculty member who is known to be a good or poor supervisor and detailed organized field notes were kept.

Also, we used the field notes to reflect emergent analytic concepts as a source of three angulations of data, frequently reconsidering the data, and referring to field notes in the context of each participant’s explanation. Analysis of the field notes facilitated in shaping contextual conditions and clarifying variations in the supervisors’ responses in each context. This led to the arrangement of several assumptions in the effect of contexts.

### Data analysis

We simultaneously performed data collection and analysis. We read the scripts carefully several times and then entered them into MAXQDA (version10). We collected and analyzed the data practically and simultaneously by using a constant comparative method. Data were analyzed based on the 3-stage coding approach, including open, axial, and selective coding by Strauss and Corbin In the open coding stage, we extracted the basic concepts or meaning units from the gathered information. Then, more general concepts were formed by grouping similar concepts into one theme. The themes became clearer throughout the interviews. Then, the constructs of them were compared with each other to form tentative categories. After that, we conducted axial coding by using the guidelines given in Corbin and Strauss’s (2008) Paradigm Model [21]. The extracted themes (codes) in the previous (open coding) stage were summarized in 3 main themes during the axial coding stage, and then the core variables were selected in the selective coding stage [20]. To generate a reasonable theory to the community, a grounded theorist needs to condense the studied happenings a the precise sequence. To check the data against categories, the researcher asks questions related to certain categories and returns to the data to seek evidence. After developing a theory, the researcher is required to confirm the theory by comparing it with existing theories found in the recently available research [21]. We finalized the model after 5 days; during this time, we explained the relations between subcategories and the core category for realizing theoretical saturation and clarifying the theoretical power of the analysis explained about work as narration.

In terms of accuracy improvement, we used the Lincoln and Guba’s criteria, including credibility, dependability, conformability, and transferability [22, 23].

To increase credibility, we collected data from different universities in Iran, and their credibility was also confirmed by three reviewers and experts in qualitative research. Also, some of the participants rechecked the data and the investigators’ description and interpretation of their experiences carefully. Prolonged engagement and tenacious observation facilitated the data credibility. In this way, the process of data collection and analysis took 12 months. Data triangulation and method triangulation also confirmed credibility [20]. The use of the maximum variation sampling method contributed to the dependability and conformability of data. Furthermore, once the explanation of the phenomenon was full, it was returned for confirmation to 3 participants of each university, and they validated the descriptions. Finally, to attain transferability, we adequately described the data in this article, so that a judgment of transferability can be made by readers.

### Ethical considerations

This study was approved by the Ethics Committee of Isfahan University of Medical Sciences (92–6746). The participants were informed about the research aim and interviews. Informed consent for conducting and recording the interview was obtained. The confidentiality of the participants’ information was maintained throughout the study.

## Results

**Fig. 1:**
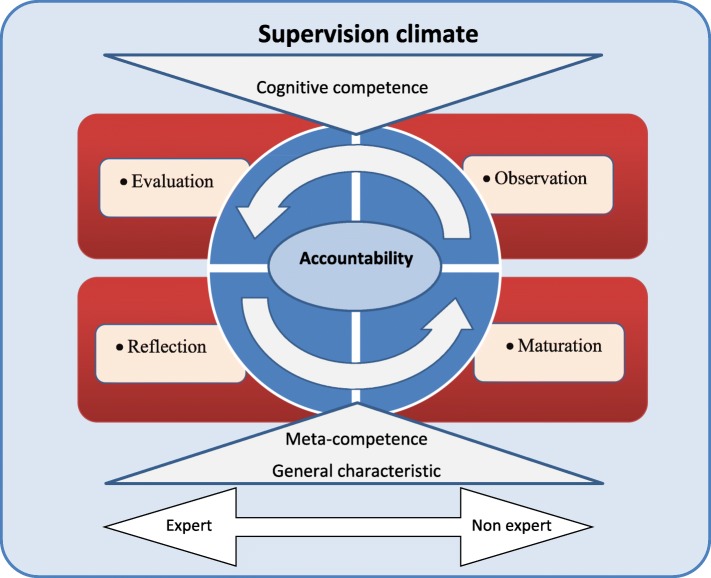
The process of expertise attainment in research supervisor model

In this study, the mean age of the faculty members and students was 44.34 ± 14.60 and 28.54 ± 2.38 years, respectively. All the faculty members and most of the students were married. Only three students were single. Three themes and seven interrelated sub-themes emerged from the data (Table 1). The main variable, which explains the process of expertise as the phenomenon of concentration and makes an association among the categories, was interactive accountability. The key dimensions of the expertise process are displayed in a model (Fig. 1).

**Table 1:** Process of expertise in research supervision: themes, sub-themes and codes

Themes	Sub themes	Code
Engagement	Curious observation	personal interest, self-awareness, meet the students’ needs, detect weaknesses in research skills, observation on role models
	Evaluation the reality	academic dignity,competition
Supervision climate	Challenging with shortcomings	Inadequate resources, work position, organizational Change, work overload, admission of students over the capacity, new rules and regulation of scholarship
	Role ambiguity	Role ambiguity in thesis supervision, sudden changes in personal life
	various performance	Ineffective evaluation, inadequate feedback
Maturation	Reflection in action	self-directed learning, participatory teaching and learning strategies learning through a hidden curriculum
	Reflection on action	Conditional Self-efficacy by expertise experience
	Interactive Accountability	Various quality attributes from expertise to in expertise

### Theme 1: engagement

In this theme, the initial phase of expertise, the supervisor starts to observe the others’ behavior in the students’ supervision and guidance based on the practical and cognitive skills previously acquired. They attempt to recognize the different needs based on the amount of their motivation and previous competence so that the models become important for them, and they recognize the scope of the needs based on their importance. Then, they try to understand the needs and values of real thesis supervision in this context. In this theme, two sub-themes, curious observation, and evaluation with reality emerged.

#### Curious observation

In this sub-theme, several concepts such as personal interest, self-awareness, ability to meet the students’ needs, ability to detect weaknesses in research skills, and observation of role models in this area act as the impellent factors in expertise attainment in research supervision.

Regarding personal interest, a successful faculty member in the area of research supervision said:

*“…In my experience, faculties must be selected from those who have curious personalities as well as being good observers, first of all. In this way, they will have the appropriate intrinsic character to acquire knowledge in guidance and supervision)…” (Faculty member N0.3)*

According to our participants, the most important intrinsic motivation is the desire to update the content knowledge and skills in research supervision. An experienced professor said:

*“*…*The knowledge gap between the new and old generations of faculty members is what forced me to update my knowledge...and it has been detected by myself…” (Faculty member N0.3).*

Another important intrinsic motivation is the ability to meet the educational and research needs of students. However, usually these needs are combined; one of the faculty members put it:

*“…I would like to be an expert in this process (thesis supervision) to meet my students’ needs. Because I have seen and felt this need many times before…” (Faculty member N0.12).*

Since the publication of research directly affects the promotion of a faculty, some professors seek skills that are practical in article publication such as several statistical and basic skills for thesis writing. The participants considered the self-awareness and consciousness elements as very important. Through consciousness, one can better understand their needs.

### Evaluation with reality

In this sub-theme, in the initial phase maintaining academic dignity and competition motivates the faculty members to obtain expertise in research supervision. At this point, the supervisor evaluates themself and their potentialities considering more precise features and acquired information (or data), so that they can find the distance between the optimal state and the existing conditions. They also evaluate the others’ potentialities in this field realistically and compete. Good supervision is then highlighted for them. Based on the supervisors’ experience, at this stage, they are seriously engaged in evaluation and competition.

Another motivation was obtaining academic and social promotion. Although the number of theses supervised by them can affect the academic promotion of supervisors, this effect is insignificant. The real motivation is maintaining academic dignity and competition amongst peers. A member of the clinical faculties stated:

*“*… *To enhance academic dignity, a faculty member should master various skills such as patient care, teaching, educational skills, and last but not least, research supervision. I got involved in research and thesis supervision because I felt I should not be left behind…”* (*Faculty member N0.17).*

At this stage, the junior supervisor tries to increase the cognitive knowledge in research supervision such as increasing specific knowledge of the discipline, planning, directing of a project effectively, and developing good interpersonal skills presented in research supervision.

### Theme 2: supervision climate

In this theme, we describe the contextual factor which changes the process of expertise attainment in thesis supervisors. The result of the study reflects some concerns about the relationship between individuals in the context in that they interact purposefully but with barriers. The supervision climate in the thesis supervision process in this theme led to the emergence of two sub-themes, challenging shortcomings and role ambiguity. These challenges include poorly structured rules and regulations which, in turn, can cause confusion and role ambiguity.

### Challenging shortcomings

This report shows that contextual factor plays a significant role in promoting the quality of a thesis in a university, but the process is faced with altered challenges such as inadequate resources, inadequate time, and ineffective evaluation and rule and regulation deficit. These challenges include the following. Most faculty members and students have experienced these shortcomings.

Various inadequate resources, such as access to new and online journals, laboratory equipment were one of the challenges for supervisors in certain aspects which required more competency, and the constraints on communication with the other academic centers worldwide undermine the sense of competition and hinder the effort put in to become an expert. One of the students said: *“… I see how difficult it is to gain access to a good article or laboratory materials in this situation …we try, but it just isn’t possible...” (Faculty member N0.17).*

Based on our results, the sudden changes in personal life, work position, and organizational change can affect the path to expertise. These changes such as marriage, work overload, admission of students over the capacity, new rules and regulation of scholar citizenship, promotion and so on can have both positive and negative impacts, depending on whether they facilitate or restrict the professional development of faculties as supervisors. For instance, an increase in student admission causes work overload, which results in neglecting self-improvement.

*“…As you know, we are over- loaded with students (they have increased the number of admissions), which is beyond our capacity. This means that most of our time will be dedicated to teaching. Self-improvement is difficult due to lack of time…” (Faculty member N0.6).*

### Role ambiguity

Poorly structured supervision can occur where there is an ambiguous context of supervision structure, supervisors and students’ roles. Most participants, as faculty members, managers, and students have experienced some difficulties in this regard, due to poorly structured rules(EDITORS NOTE; do you mean ‘rules and regulations ‘here) and regulations and its impact on the thesis supervision. It is not only the rules themselves but also the way they are implemented. One of the faculty members expressed confusion over the rules related to the dissertation as follows:

*“…It should be made clear what I must do exactly. It is obvious regarding supervision on the work of students; there are not the same expectations from an Assistant Professor, Associate Professor, and a professor. Most problems occur as a result of the gap in legislation; For example, the rules imply a full Professor does not need a statistical consult, while many supervisors like me do not have enough knowledge and skills in statistical analysis...” (Faculty member N0.1).*

Failure to implement the rules also increases the sense of this ambiguity, and there are no specific rules for verifying capability and audits to determine inadequate experts in thesis supervision. The role ambiguity or unclear roles and responsibilities of the supervisor and student in the thesis process were other limitations that were emphasized by the majority of participants. A faculty member stated:

*“… Supervisors have different roles during the thesis process. To enhance this process, one must exactly know one’s responsibilities. For instance, in the beginning, the supervisor should guide the students through the process of finding a suitable research topic, but if the teacher's role is unclear, then instead of guiding they may actually choose the topic, and if so, the students will be prevented from exploring, using their creative thinking, and improving their problem-solving abilities…” (Faculty member N0.1).*

### Various performance

Based on the participants’ experiences, in this situation in which there are inadequate resources and organizational and social problems, some faculty members are well-trained in the field of supervision. One of the senior faculty members said: *“It is my honor to mention that despite the existence of many obstacles, I have been able to train well-educated students, who have become researchers and contribute to the development of science in my country.”*

One of the most important causes of poor performance is ineffective evaluation. Based on the participants experiences, two main problems can result in ineffective evaluation. First of all is the inadequate feedback from the supervisor which leads to unmotivated learners and the second one is lack of feedback from the stakeholders and educational institutes which in turn diminishes the supervisor’s efforts toward self-improvement. These can lead to poor performance both in students and supervisors.

In one of the Ph.D. student’s words:

*“…In this system, there is no supervision on the supervisors; there is no control or evaluation of their work. Also, the supervisors don't get feedback from their students during the research process, and there is no third person who investigates whether the report is real or not…” (student N0. 7).*

Evidence from data suggests that an unfair judgment and evaluation of academic theses are other problems in the process of acquiring the merit of teachers. If there isn’t proper evaluation, students and supervisors would not have the right standards to correct their performance.

The professors do not always consider the lack of expertise to be the only cause of poor performance. Many believe that inadequate monitoring can also reduce the motivation for quality performance. This means that supervisors may obtain the necessary expertise, but they are not motivated to enhance their performance since they are not expected to do this. One student had experienced:

*“…I was so thrilled that my thesis supervisor was an experienced, older and well-known professor, but unfortunately, I soon found out that not only was his scientific knowledge outdated, but also he lacked the necessary supervision skills, so he let the students do all the work unsupervised. He did not take any responsibility during the process…”* (Student N0.4).

Another point which leads to poor performance is the fact that some faculty members do not comprehend the main purpose of the thesis writing process; actually, they do not know the difference between teaching and guiding in the project or thesis supervision. One of the basic science supervisors said:*“… Some faculties consider a thesis as research work and not a lesson in which research methodology should be taught...” (Faculty member N0.5).*

Performing poorly along with ignoring professional ethics can also lead to increased tension and stress in student-teacher relationships. This can result in despondency and frustration in both students and teachers and create a vicious cycle of inefficient supervisors who will train inefficient students or future supervisors.

One of the students put it this way:

*“...I feel the absence of a supervisor in my research; I would have been more successful, and my results would have been better if I had had more guidance.” (Student N0.6).*

### Theme 3: maturation

In this theme, the secondary phase of expertise, the individual is emotionally involved and feels that success or failure is important. This is a stage in which the learner needs an integrated schedule to be competent, and as a result, success or failure will follow. The supervisors frequently think about personal promotion and takes action in this way. They try out different approaches, and sometimes due to disappointment and embarrassment they fail. Some individuals quit at this stage and never reach competence, or they have what may be called an artificial competence. And this does not mean that they are not considered to be well-known supervisors; rather, they know, as do the students, that they are not competent. At this stage, the supervisor attempts to acquire the identity of a researcher and tries to enhance his availability, and be dutiful, knowledgeable, and enthusiastic in research supervision. Along the lines of this theme, three sub-themes of Reflection in action, Reflection on action, and Interactive accountability emerged.

### Reflection in action

In this sub-theme, the patterns of expertise development begin, and self-directed learning, participatory teaching and learning strategies through a hidden curriculum are considered. At this stage, the supervisor tries to follow self-directed learning, and the amount of time allocated to expertise acquirement seems to be one of the most important factors. In this regard, one stated:

*“…My success in this case (research supervision) is, first of all, due to self-evaluation and self-effort. For instance, to be in control and take full responsibility, I think about everything related to the guidance of the students, and I felt the need to master every aspect of research, even the statistical skills needed for analysis…” (Faculty member N0.8).*

The supervisors’ activities were divided into two groups: self-directed –learning strategy and gaining experience through individual effort. Expertise requires continuous interaction and experience. They evaluate their learning, and by this, they experience the manner of managing and allocating time for effective supervision. According to participants, the amount of time allocation for expertise seems to be one of the most important factors for self-directed learning and expertise acquirement.

The formal training workshops provided an opportunity for supervisors with similar terms and the same problems in terms of learning experiences, environmental features, students, and educational problems to come together in one place. Participants also considered the formal participatory teaching necessary since it can provide an opportunity for the peers to get together and exchange their experiences. As a clinical faculty member put it:

*“…Collaborative strategies can be beneficial in many ways. One of them is the facilitation of experience exchanges amongst teachers, peers, and colleagues and modeling the behavior of teachers and teaching workshops that emphasize the importance of their expertise in research supervision…” (Faculty member N0.1).*

In our participants’ experience, this self-directed learning is effective if, and only if, it is done accompanied by proper training and participatory teaching. Otherwise, it is a waste of time. As an example, one of the students in this field said:

*“…my supervisor was a great teacher and put in a lot of time and effort on my thesis supervision; however, due to his lack of research skills, I had to change my thesis proposal three times. However, after he* participated in a training course at the University of Oxford, *his progress was unbelievable and impressive…and I saw his expertise…” (Student N0.11).*

One of the faculty members also quoted:

*“…When the teachers feel a gap in their knowledge or skill, the university must provide a comfortable, appropriate, and easy way for learning them …” (Faculty member N0.10).*

Regarding this subject, one of the Managers in this field stated:

*“…Another improvement strategy is the use of interpersonal interactions among faculty members, these instructive interpersonal interactions among the faculty members in similar conditions make it possible to benefit from peers’ feedback …” (Manager N0.1).*

A hidden curriculum strategy, like learning through trial and error can also affect the expertise process. One of the professors expressed:

*“… Learning through trial and error is very effective; through the supervision of each thesis, we learn some of our mistakes and try not to remake them in the next one …” (Faculty member N0.3).*

The professors do not always consider the lack of expertise to be the only cause of poor performance. Many believe that inadequate monitoring can also reduce the motivation for quality performance. This means that supervisors may obtain the necessary expertise, but they are not motivated to enhance their performance since they are not expected to do this. One student’s experience:

*“…I was so thrilled that my thesis supervisor was an experienced, older and well-known professor, but unfortunately, I soon found out that not only was his scientific knowledge outdated, but also he lacked the necessary supervision skills, so he let the students do all the work unsupervised. He did not take any responsibility during the process…” (Student N0.4).*

### Reflection on action

The learner provides an integrated schedule for their competence and uses all the facilitators and facilities around them for further efficiency and promotion. This stage is named Conditional Self-efficacy by expertise experience. At this stage, the supervisor is considered a competent individual who can guide the students based on the experiences of specialized and non-specialized faculty members.

In this regard, one of the students said:

*“…I can acknowledge that my supervisor functioned very impressively in this thesis, but guidance and supervision are not static; rather, it is an active process. To be a good supervisor, the faculty members should try to keep up to date and revise their attitudes, duties, and their specialty and knowledge. …” (Student N0.3).*

According to the participants, at this stage the supervisors have achieved meta-competence and general characteristics or professional value; are able to guide the students and others; and develop characteristics such as acquiring specific knowledge of the discipline, especially well-organized knowledge, planning, directing of a project effectively, having good interpersonal skills, and being dutiful, knowledgeable and enthusiastic in research.

One of the PhD students states: *“… My supervisor is typical of an expert. His ingenious inquiries, extraordinary attention to science and his personality have always been admired and he has been a role model for me…” (Student N0.6).*

For example, the supervisors attend educational programs on scientific writing and thesis evaluation as well as ethics in research and apply them in team work. Gradually, their competency can enable them to function as a good supervisor for their students. At this stage, the supervisor develops so that they can respond due to discovery and intuition. These responses replace their dubious and unskilled reactions. The supervisor now reflects various stages of supervision and guidance. They take action, and in fact, a part of their reactions are achieved through observation and recognition. In this stage, they not only recognize what should be done but also distinguish how to achieve it with more precise discretion. A competent person does the appropriate task in the most appropriate time using the right platform.

The time period required for training or acquiring expertise varies from one person to another. Some individuals become experts very soon, whilst it takes others longer.. As one of the professors said:

*“…In the beginning, I was too concerned with my responsibility as a thesis supervisor and was not sure what I should do. However, after ten years of experience, I have gained a sense of awareness which makes supervision easier for me. Of course, up to date knowledge and skill as to managing a thesis are always necessary. It took me about 12 years to reach where I am today. Furthermore, an individual who is expert at present, will not be so in two years, so I want to say that the expertise in thesis supervision in a continuum, which depends on the supervisor’s reflections on work and activity …” (Faculty member N0.15).*

The continuous path of expertise in supervision can be affected by various factors. This has resulted in a range of expertise and performance in supervisors. This range and continuum is a theme that most of our participants agreed with. One of the managers revealed:

*“…There is surely a continuum of expertise. We cannot deny the expert supervisors; however, the existence of those with poor supervising skills must also be acknowledged (in thesis supervision). There are those on whose ethics, honesty, and knowledge we can rely on. On the other hand, there are a few who are not as trustworthy as needed.” (Manager N0.1).*

### The core variable: interactive accountability

As shown in Fig. 1, through this survey, we found that the core variable in thesis supervision process is the interactive accountability shaped by interactions of supervisors and students in an academic setting, so to enhance the accountability, each group must take responsibility and do his or her job. In this regard, one of the managers claimed:

*“…When supervisors find themselves responsible, and the university officials recognize this responsibility, the supervisors are motivated to seek expertise and try to enhance their competencies and acquire learning strategies because of being accountable…” (Manager N0.2)*

This means that teachers must be responsive to the needs of students, university and community. Accountability is a mutual interaction between the students and their supervisor, in other words, if the student is responsive to his duties, he creates motivation in his supervisor. One of the participants commented;

*“…I've always tried to be a competent thesis supervisor, so that I have the ability to meet the needs of the community and university as well as students. I say to myself when I accept the supervision of a thesis, I should be well accountable for its results…” (Faculty member N0.32)*

## Discussion

This study aimed at exploring the processes of expertise among thesis supervisors based on the experience of faculty members, students, and managers of Iranian universities of medical sciences. The section concludes with an explanation of how these themes are a cohesive relationship, which enables the expertise development of supervisors. It seems that the core variable in the expertise process is the concept of interactive accountability and efforts to acquire the capacity to respond to the students and academic needs. This will help them to promote their professional behavior in research supervision. The importance of accountability and various types of ability in thesis supervision has also been emphasized by other studies [24–26]. It was also mentioned as the major feature of the supervisor in other studies [26, 27].

In this study, “accountability” emerged as the behavioral pattern through which the supervisors resolved their main concern of being an expert in being responsive to academic and students’ needs. Supervision training is complex since academic choices in the real world can depend on supervisor characteristics. The results of this study revealed that in the initial phase of supervision, observation, evaluation, and reflection in action and maturation stage in the secondary phase were the major themes that emerged. This result compared with Bandura’s social learning and self-efficacy theory was significant in similarity and difference. Bandura believes that achieving self-efficacy is one of the most important contributors to competence. In his model, he suggested four sources of self-efficacy, including previous accomplishments, vicarious experiences such as having a role model, verbal persuasion such as coaching and evaluative feedback, and emotional arousal [28, 29]. Likewise, in this study, we found that the emotional arousals such as personal interest in cooperative learning, peer competition, meeting the needs of students, self-awareness and the need for upgrading are the significant factors for the faculties’ expertise. Also, our participants found that the utilization of previous experiences is the most effective method of achieving personal competence. However, this study indicates conditional expertise, which means if an expert’s information is not up to date and they do not make any effort in this regard, being an expert and having expertise is not a permanent condition.

This study also revealed that self-effort, workshops, and role models, as part of a hidden curriculum, are influential methods of teacher empowerment which agrees with the results of some studies such as those of Britzman et al. and Patel et al. Patel et al. have also suggested the importance of role modeling; they believe that modeling and observing other faculty members behavior is an effective tool for promoting and strengthening the sense of efficacy in learners [30, 31].

Based on our study results, among the learning methods used in Iran, the collaborative education and problem-based learning is the widely accepted method which is preferred by most faculties. Therefore, cooperative and collaborative learning strategies can be used in educating the faculty members towards expertise in supervision, as revealed in other studies [32, 33].

Lack of time is reported by supervisors to be one of the most common barriers in trying to become an expert and carry out respectable worthy supervision, and taking one’s time is acknowledged as a motivating factor for putting in more effort in thesis supervision [34–36].

The effect of contextual factors is studied in several surveys [36–38]. Gillet et al. state that contextual and organizational factors play a key role in the competence of teachers in research supervision [36]. This study also showed that faculty expertise in thesis supervision was significantly affected by the impact of contextual interventional factors such as sudden changes, structural shortcomings, and educational environment. Based on our and other studies’ results, among the sudden changes, increased workload due to the increase in the student population has greatly affected expertise. Moreover, while an increase in the workload can lead to more experienced faculty members, it is very time-consuming and, therefore, reduces the chance to obtain new information and skills in thesis supervision [33, 37].

Similar to our study, other studies such as those of Al-Naggar et al. and Yousefi et al. have also found insufficient monitoring and lack of formative evaluations to be one of the main obstacles in the thesis supervision process. Studies have indicated that to improve the supervision process, careful planning and incentive rules must be applied [5, 34]. Similarly, our participants mentioned that rules and regulations which have resulted in the positive effect of research on scholarship and promotion had truly motivated them. Like our study, other studies in Iran have also found that the amount of time allocated to learning is one of the influential factors affecting the faculty members’ expertise [13, 38]. A malfunctioning relationship between the student and supervisors can affect both of them negatively; that is, it can compel the students to misbehave and also reduce the teachers’ motivation to develop better skills. This malfunction may be due to the lack of constructive interactions or paternalism leadership in research supervision [39, 40]. As shown in Fig. 1, this study provided a conceptual framework that can be used in policy making and studies of expertise development in research supervision. This framework is based on the perception and experience of the majority of those involved in the thesis process. It also provides teachers with an opportunity to compare and share their experiences.

This model has three fields of experience, which yields a comprehensive gradient of the factors used for the development and progress of thesis supervision quality. In other words, it is a rational structure that makes an effort to cover a comprehensible number of stages, of concept, achievement, and impact or consequence. In other words, this model is a combination of a great number of items that help to recognize the present and future processes of expertise in thesis supervision, and future challenges in this area which predict results and impacts of supervisor’s knowledge, attitude and research supervision. Table one offers the categories and clarifications [17].

This study is based on our overall model of expertise attainment. This model reveals that specific personal efforts such as observation of prior knowledge, evaluation or self-assessments alongside the university contextual dynamics help to figure out how supervisors select their approaches and engagements, and respond carefully to their task, which in turn impacts the supervisors’ level of expertise and, finally, outcomes such as work and perseverance, which then help them to become an expert. Similar to the social learning theory of Bandura, this model also states that there is a mutual relationship between different parts that can mutually affect one another. For instance, faculty members have shown in various studies how one’s previous academic success and failure can affect the future levels of involvement and motivation. Based on the study aims, we focused on only three of the components of the model: observation, evaluation, and self-efficacy; in terms of motivational processes, we focused on four motivational components. The first is self-efficacy, defined as students’ judgments of supervisor abilities to carry out a task, and their beliefs about their ability to do so show the highest levels of academic achievement and also engagement in academic behaviors promoting learning.

Through the use of this grounded theory, we can begin to understand the supervisors’ challenges and why it may be difficult to become an expert in research supervision in practice. The junior supervisors curiously observe and evaluate their environment by reflection and in action and do their best to attain knowledge and skills in the supervision of the theses, so that they can reach maturation. They are mainly supported by prior knowledge of the research supervision, which they had acquired when they were students. The concept of “interactive accountability” refers to the fact that if the supervisor is responsive to the students’ needs, they can be an expert in supervision. If they cannot overcome the barriers and shortcomings such as lack of time, they will not attain expertise in thesis supervision.

### Strengths and limitations of the study

This grounded theory study describes the main dimensions of expertise in research supervision from straight reports of a large qualitative sample (*n* = 84) which consists of thesis supervisors, from all Iranian universities in three different data collection phases. Like other qualitative research, the results of this study cannot be generalized; therefore, it is recommended that the researchers conduct further qualitative research in other contexts to support these findings.

Despite the above limitations, we believe that this model can be useful for supervisors in the thesis supervision area, not only in analyzing the supervisors’ experience of supervision and being an expert but also in recognizing the areas of intervention or development of teacher training.

### Implications of the study

The findings of the present study will help administrators to choose the supervisor with definite criteria in medical sciences institutes and facilitate the expertise in the supervision process through elimination of the shortcomings and improvement of the educational climate. The supervisor’s interest, talent, and capabilities should be assessed at the beginning of their employment as academic staff. Supervisors should attend educational workshops for updating their knowledge about supervision. It is recommended that collaborative strategies and methods should be used, so that we can contribute to the process of becoming an expert. The assessment of supervisors’ functioning in supervising and provision of feedback can contribute to the process of expertise. Feedback received from students about their supervisors will improve the supervisor’s further expertise and capabilities. For future studies survey on the impact of successful models in thesis supervision, disclosure analysis studies about student and supervisor are recommended.

## Conclusion

In this study, we aimed to find out how thesis supervisors achieve expertise in supervision. The results of our study indicated that thesis supervisors achieve expertise in supervision in two stages of engagement and maturation. The emotional need to be responsive towards peers and students is the main motivation for the acquisition of competency at observation and evaluation phase of engagement. Through the evaluation and observation phase, the supervisors reach cognitive competence, such as research skills. Also, in the maturation phases, they reach meta-competence in research supervision such as problem-solving and resolving dilemmas by reflection in and when exposed to dilemmas. Meanwhile, the effects of supervision climate include shortcomings and role ambiguities which should be taken into account. According to this model, when supervisors are exposed to such problems, they apply multiple strategies, such as self-directed and collaborative learning; and learning by trial and error and from the role models. This will help them to promote their professional behavior in research supervision. This study indicated that interactive accountability, as the core variable, can be guaranteed in thesis supervisors by making the role clear, creating a supportive context, and improving the academic competencies of staff in an ongoing fashion. Therefore, this can promote constructive expertise in supervisors and foster a deeper understanding of the supervisor’s expertise in thesis supervision.

## Data Availability

The datasets produced and analyzed during the present study are not publicly accessible due to participant confidentiality, but are obtainable from the corresponding author on reasonable request.
